# Recurrent CNVs in constitutional CMA: a ClinGen-anchored single-center postnatal and prenatal experience

**DOI:** 10.3389/fgene.2026.1960791

**Published:** 2026-10-20

**Authors:** Nihan Bilge Satkın, Engin Atlı, Sinem Yalcintepe, Kübra Müge Çelik, Hakan Gürkan

**Affiliations:** 1 Department of Genetics and Bioengineering, Faculty of Engineering, Trakya University, Edirne, Türkiye; 2 Department of Medical Genetics, Faculty of Medicine, Trakya University, Edirne, Türkiye

**Keywords:** chromosomal microarray analysis, ClinGen dosage sensitivity map, constitutional cytogenomics, copy-number variation, dosage sensitivity, postnatal diagnosis, prenatal diagnosis, recurrent genomic disorder regions

## Abstract

**Background:**

Recurrent copy-number variants (CNVs) mediated by low-copy repeats or segmental duplications constitute an important subset of constitutional chromosomal microarray analysis (CMA) findings. Although ACMG/ClinGen standards have improved CNV classification, the burden of recurrent CNVs is not routinely quantified as a canonical, interval-matched, direction-specific category in clinical CMA output. We applied a strict ClinGen-anchored framework to quantify recurrent genomic disorder intervals in a consecutive single-center CMA series.

**Methods:**

Constitutional array-CGH and SNP-array records from a single center (2014–2026) were retrospectively reviewed, including 1,661 postnatal probands and 401 prenatal samples, with aneuploidies excluded. Recurrent regions were defined using the ClinGen Dosage Sensitivity Map and restricted to canonical intervals presumed to be mediated by non-allelic homologous recombination. Assignment required breakpoint concordance with the flanking low-copy repeat blocks and canonical interval size; terminal, unbalanced, and atypical imbalances were excluded. All records were re-curated against a single ClinGen release, with earlier GRCh37 coordinates converted to GRCh38.

**Results:**

Pathogenic or likely pathogenic findings were identified in 278/1,661 postnatal probands (16.74%) and 28/401 prenatal samples (6.98%). Recurrent CNVs were detected in 92/1,661 postnatal (5.54%, 95% CI 4.54–6.74) and 17/401 prenatal cases (4.24%, 95% CI 2.66–6.68). Pathogenic/likely pathogenic CNVs were found in 66/92 postnatal (71.74%) and 7/17 prenatal carriers (41.18%); the remaining findings were of uncertain significance or benign. Pathogenic recurrent CNVs thus accounted for 66/278 (23.74%) of postnatal and 7/28 (25.00%) of prenatal non-aneuploid pathogenic findings. Proximal 22q11.21 and 16p11.2 were most frequent. Deletions predominated postnatally (64/93, 68.82%); duplications comprised 11/17 (64.71%) of prenatal recurrent CNVs. Postnatal pathogenic recurrent-CNV yield was 58/1,589 (3.65%) in phenotype-driven testing (no suspected recurrent-CNV syndrome) and 6/19 (31.58%) with syndrome-specific suspicion; neurodevelopmental referrals contributed most cases.

**Conclusion:**

ClinGen-defined recurrent CNVs were a substantial component of constitutional CMA findings. However, only about three-quarters of postnatal and fewer than half of prenatal carriers harbored pathogenic/likely pathogenic CNVs, indicating that a recurrent-region label alone is insufficient for interpretation. These findings support region- and dosage-specific reporting, with evidence-based classification distinguished from clinical interpretation, particularly for prenatal findings and inherited duplications, where the same label may have different patient-specific implications.

## 1 Introduction

Copy-number variants (CNVs) are a key molecular cause of intellectual disability, autism, congenital malformations, and pregnancy loss. Chromosomal microarray analysis (CMA), which identifies submicroscopic CNVs across the genome, is the primary cytogenomic test recommended for these indications in the postnatal setting. The same first-tier recommendation also applies prenatally for fetuses with significant structural anomalies detected on ultrasound and for assessing intrauterine fetal loss and stillbirth when cytogenetic testing is performed (Vissers et al., 2016; Shaikh, 2017; Shao et al., 2021).

Many clinically important constitutional CNVs occur at recurrent genomic disorder loci that are typically formed by flanking low-copy repeats or segmental duplications, which predispose to non-allelic homologous recombination (NAHR)-mediated deletions and reciprocal duplications with clustered or broadly shared breakpoints. These recurrent CNVs are well recognized in neurodevelopmental and congenital disease settings, but their clinical consequences are highly locus- and dosage-dependent, with variable expressivity and incomplete penetrance across regions and between reciprocal deletion and duplication states (Dittwald et al., 2013; Gillentine et al., 2018; Harel and Lupski, 2018; Smajlagić et al., 2021). This asymmetry complicates clinical interpretation and requires the two reciprocal dosage states to be evaluated separately, a distinction especially critical in the prenatal context, where low-penetrance variants are detected before phenotypic manifestation and are frequently inherited, even in the face of variable expressivity (Rosenfeld et al., 2013; Maya et al., 2018). Recent prenatal alignment work also highlights persistent variability in how low-penetrance ClinGen recurrent CNVs are classified and reported across laboratories (Shear et al., 2025; Soares et al., 2026).

These variants are individually rare but collectively common: in an unselected newborn cohort, recurrent neurodevelopmental CNVs had a cumulative prevalence of 0.48% (∼1 in 200 live-born children) (Smajlagić et al., 2021). Their clinical burden is locus-specific, with population and biobank studies showing variable prevalence across regions and broad phenotypic associations with neurodevelopmental and medical outcomes (Smajlagić et al., 2021; Birnbaum et al., 2022).

ClinGen dosage curation and ACMG/ClinGen CNV interpretation standards provide a framework for more consistent CNV classification and reporting (Riggs et al., 2018; 2020). Several studies have already used ClinGen or ClinGen-listed recurrent loci in related ways: Kang et al. quantified pathogenic recurrent CNVs in a prenatal CMA cohort using ClinGen HI/TS-based region selection, Xue et al. systematically classified 22q11.2 recurrent CNVs using ClinGen curation, and broader population studies have estimated prevalence or risk for predefined recurrent CNV loci (Gillentine et al., 2018; Smajlagić et al., 2021; Xue et al., 2021; Kang et al., 2024; Vaez et al., 2024). However, prior approaches have generally been region-specific, phenotype-specific, population-based, or based on selected recurrent-locus sets, rather than on a consecutive series of diagnostic CMA evaluations against canonical ClinGen-curated recurrent intervals. In addition, studies using ClinGen in this area have typically relied on dosage-sensitivity–based locus selection, overlap-based inclusion, or single-region curation, rather than restricting the recurrent category to CNVs that match established recurrent interval boundaries and dosage direction. Therefore, this study aimed to quantify canonical ClinGen-defined recurrent genomic disorder intervals in a consecutive single-center constitutional CMA series and to distinguish their pathogenic fraction from variants of uncertain significance, while preserving prenatal/postnatal context, referral indication, and deletion/duplication direction.

## 2 Materials and methods

### 2.1 Study design and setting

This retrospective review analyzed constitutional array-CGH and SNP-array data from the Medical Genetics Department at Trakya University. Only cases that underwent CMA were included in the study. The overall laboratory archive covered June 2014-May 2026; postnatal records were available from 2014 onward, whereas prenatal CMA records were available from 2016 onward. The postnatal cohort included 1,661 unique probands (parental/repeat samples excluded); the prenatal cohort included 401 fetal samples (amniocentesis, CVS, cordocentesis). All aneuploidies were excluded. The analysis focused solely on subchromosomal CNVs, classified according to the American College of Medical Genetics and Genomics (ACMG)/Clinical Genome Resource (ClinGen) guidelines (Riggs et al., 2020). The full ACMG/ClinGen curation of recurrent CNVs is provided in Supplementary Table S2. Pathogenic/likely pathogenic findings were counted as primary anomalies; VUS were reported separately. Ethics approval was obtained (TÜTF-GOBAEK 2026/170) and written informed consent was provided. A subset of the patients included in this cohort has been reported previously in the context of different research questions and phenotypic or cytogenetic analyses (Gürkan et al., 2020; Atli et al., 2022a; 2022b; Yalcintepe et al., 2022).

### 2.2 DNA isolation and CMA analysis

Genomic DNA was extracted from amniotic fluid and chorionic villi using the QIAamp DNA Mini Kit (Qiagen, Hilden, Germany), and from cord blood and peripheral blood using the QIAamp DNA Blood Midi Kit (Qiagen, Hilden, Germany), following the manufacturers’ protocols. CMA was performed using a 180K CGH + SNP oligonucleotide array (ISCA design, Agilent, Santa Clara, CA, United States) until January 2024; thereafter, the CytoScan HT-CMA 96F Assay (Thermo Fisher Scientific, Waltham, MA, United States) was used. Data were analyzed with CytoGenomics 2.7 (Agilent) and Chromosome Analysis Suite 4.5.0.34 (Thermo Fisher), respectively, according to standard manufacturer-recommended workflows.

All CNV calls generated by the software were manually reviewed. CNVs were considered for further analysis if they met at least one of the following criteria: (i) size ≥100 kb, (ii) involved ≥20 consecutive probes, or (iii) overlapped a known disease-associated gene or recurrent region, regardless of size or probe count.

### 2.3 Recurrent CNV definition, assignment and classification

Recurrent CNV regions were defined using the ClinGen Dosage Sensitivity Map (Region Curation Results, 28 April 2026; GRCh38/hg38), which comprises 48 curated recurrent genomic disorder regions (Riggs et al., 2018; 2020). A reference list of all 48 ClinGen-curated recurrent regions, including ISCA identifiers, GRCh38 coordinates, ClinGen haploinsufficiency/triplosensitivity scores, the short region labels used throughout this manuscript, and pooled penetrance estimates from (Goh et al., 2025) where a corresponding region was analyzed, is provided in Supplementary Table S1; case-level data are given in Supplementary Table S2.

CNV region assignment followed a predefined stepwise procedure. First, overlap with a ClinGen-curated recurrent region was screened. Second, CNVs were assigned when their array-defined boundaries were compatible with the flanking low-copy repeat (LCR) blocks and their size was consistent with the canonical interval, taking account of platform resolution, probe coverage, and coordinate conversion. Minor endpoint differences were accepted when they remained consistent with the same canonical LCR-defined interval; exact coordinate matching was not required. Third, substantially larger or smaller, terminal, complex, unbalanced, or merely overlapping CNVs were excluded unless they matched a recognized ClinGen interval; overlap with a ClinGen locus or dosage-sensitive gene alone was not sufficient. Fourth, adjacent or nested intervals were resolved using ISCA/ClinGen region names and GRCh38 coordinates (Supplementary Table S1). Since recurrent genomic disorder regions arise from non-allelic homologous recombination between fixed flanking LCRs, breakpoints cluster within defined LCR blocks (Carvalho and Lupski, 2016); assignment therefore used breakpoint concordance and size consistency at array resolution rather than a percentage-overlap cut-off. Flanking low-copy repeat block boundaries were taken from the ClinGen recurrent CNV track (V2.1, GRCh38). All assignments were re-curated against the current ClinGen release and coordinates from earlier GRCh37 records were converted to GRCh38, ensuring a single reference framework for the entire series.

Region assignment and pathogenicity classification were treated as separate steps. Each recurrent CNV was classified independently under the 2019 ACMG/ClinGen technical standards (Riggs et al., 2020) as pathogenic/likely pathogenic, of uncertain significance, or benign, with deletions scored against haploinsufficiency and duplications against triplosensitivity evidence for the same region. We applied Section 5 (inheritance) when parental data were available, but it did not change the classification in any case. We recorded inheritance information descriptively, and for these recurrent CNVs, we determined the final category using Sections 2 (overlap with established dosage-sensitive regions) and 3 (number of protein-coding genes in the interval) rather than the inheritance pattern. Dosage scores, evidence codes, and point scores for every recurrent CNV are given in Supplementary Table S2. Yield estimates were calculated per carrier rather than per event, since a small number of carriers harbored more than one recurrent CNV.

### 2.4 Referral indication and test purpose

Referral indications were categorized as postnatal (neurodevelopmental/neurological disorders; congenital, dysmorphic and sensory anomalies; growth and head-size disorders; syndromic and familial indications; reproductive and sexual development disorders; and other systemic/miscellaneous indications) and prenatal (abnormal ultrasound, advanced maternal age, positive screening, other). Test purpose was stratified as targeted, confirmatory, characterizing, or diagnostic; the diagnostic stratum (phenotype-driven testing without prior suspicion of a specific recurrent CNV syndrome) served as the denominator for phenotype-driven yield estimates.

### 2.5 Statistical analysis

Proportions are reported with 95% Wilson confidence intervals, and estimates from strata with small denominators should be read together with these intervals rather than in isolation. The postnatal and prenatal cohorts differed in size, study period, and ascertainment and were described in parallel rather than compared directly; all comparisons are descriptive and not corrected for multiple testing.

## 3 Results

### 3.1 Cohort characteristics

We distinguished recurrent-CNV carriers (n = 109) from recurrent-region observations/events (n = 110); one postnatal carrier harbored two independent recurrent CNVs, both documented in Supplementary Table S2 and included in event-based regional analyses. All yield estimates were calculated per carrier. A pathogenic or likely pathogenic copy-number finding was identified in 278/1,661 postnatal probands (16.74%, 95% CI 15.02–18.61) and 28/401 prenatal samples (6.98%, 95% CI 4.87–9.91); variants of uncertain significance were identified in 58/1,661 postnatal probands (3.49%) and 23/401 prenatal samples (5.74%), with recurrent-region findings in 22/58 (37.93%) and 8/23 (34.78%), respectively. Recurrent CNVs were identified in 92/1,661 postnatal probands (5.54%, 95% CI 4.54–6.74) and 17/401 prenatal samples (4.24%, 95% CI 2.66–6.68). Pathogenic/likely pathogenic CNVs were found in 66/92 postnatal (71.74%) and 7/17 prenatal carriers (41.18%); the remaining findings were of uncertain significance or benign. Pathogenic recurrent CNVs therefore represented 66/278 (23.74%) of postnatal and 7/28 (25.00%) of prenatal non-aneuploid pathogenic findings, corresponding to 66/1,661 postnatal probands (3.97%) and 7/401 prenatal samples (1.75%). Fetal ultrasound anomaly was the primary indication in 348/401 prenatal cases (86.78%). Among fetuses referred for an ultrasound anomaly, 12/348 (3.45%) carried a recurrent CNV and 6/348 (1.72%) carried a pathogenic recurrent CNV. Table 1 summarizes cohort characteristics, and Figure 1 shows the study flowchart.

**TABLE 1:** Characteristics of the postnatal and prenatal cohorts.

Characteristic	Postnatal	Prenatal
Probands/samples, n	1,661	401
Study period	2014–2026	2016–2026
Pathogenic finding (non-aneuploidy), n (%, 95% CI)	278 (16.74, 15.02–18.61)	28 (6.98, 4.87–9.91**)**
Recurrent CNV carriers, n (%, 95% CI)	92 (5.54, 4.54–6.74)	17 (4.24, 2.66–6.68)
Recurrent, pathogenic/likely pathogenic, n	66	7
Pathogenic recurrent, % of all pathogenic findings	23.74%	25.00%
Distinct ClinGen regions (all classifications), n	23	9
Deletion-only/duplication-only recurrent-CNV carriers, n*	63: 28	6: 11
Inheritance documented, n (%)	73/92 (79.35)	17/17 (100)
*de novo*/maternal/paternal	38/23/12	6/7/4
Transmitting parent with a documented clinical diagnosis, n	1/35	0/11

* One postnatal carrier (P025) had both a recurrent deletion and a recurrent duplication and is excluded only from this dosage-direction breakdown, not from the cohort totals. Both CNVs, are included in the event-level counts in Table 2 and Supplementary Table S2.

**FIGURE 1:**
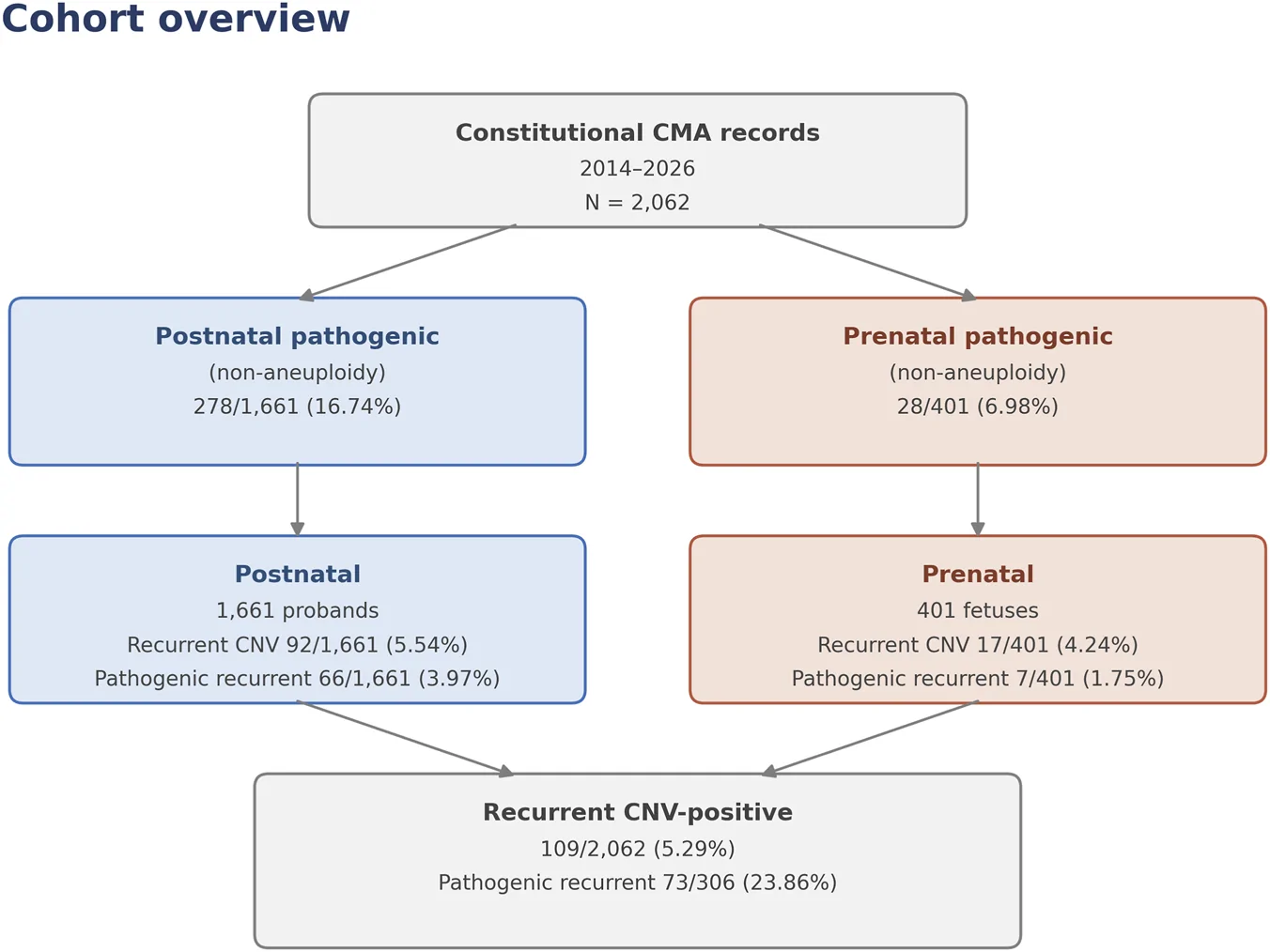
Cohort selection and recurrent CNV burden.

Region- and direction-specific counts, along with their ACMG/ClinGen classifications, are given in Table 2. The most frequent postnatal entities were 22q11.21 proximal deletion (DiGeorge syndrome), 16p11.2 proximal (BP4–BP5), Xp22.31 (*STS*), 7q11.23 proximal (Williams–Beuren), 17q12 (*HNF1B*; renal cysts and diabetes), and 15q11.2–q13.1 (Prader–Willi/Angelman). All 1q21.1 carriers fell within the distal (BP3–BP4, *GJA5*) subregion; 7q11.23 proximal (Williams–Beuren) and 17q21.31 (*KANSL1*; Koolen–de Vries) were observed only in postnatal cases. In one postnatal carrier, the co-occurring second lesion was itself a recurrent region, so that this patient carried two independent recurrent loci (10q11.22–q11.23 duplication +15q11.2 BP1–BP2 deletion). Of the 46 inherited CNV carriers (35 postnatal, 11 prenatal), one transmitting parent had a documented diagnosis (Table 1). All 11 prenatal transmitting parents were recorded as clinically unaffected; postnatal parental status was derived from referral records.

**TABLE 2:** Event-level recurrent CNVs by ClinGen region, dosage sensitivity, dosage direction, and ACMG/ClinGen classification in postnatal and prenatal cases.

Recurrent region	HI	TS	Post del	Post dup	Pre del	Pre dup	Total	Deletion	Duplication
22q11.21 proximal (A–D, *TBX1*)	3	3	13	5	1	·	19	P	P
16p11.2 proximal (BP4–BP5, *TBX6*)	3	3	9	2	1	·	12	P	P
Xp22.31 (*STS*)	3	40	3	3	1	2	9	P	B
1q21.1–q21.2 distal (BP3–BP4, *GJA5*)	3	2	3	2	1	3	9	P	VUS
7q11.23 proximal (*ELN*, Williams–Beuren)	3	3	5	2	·	·	7	P	P
17q12 (*HNF1B*)	3	3	4	1	1	·	6	P	P
15q11.2 (BP1–BP2, *NIPA1*)	3	40	4	1	1	·	6	P	B
15q11.2–q13.1 (class II, BP2–BP3, PWS/AS)	3	3	6	·	·	·	6	P	—
16p13.11 (BP2–BP3, *MYH11*)	2	2	1	4	·	·	5	VUS	VUS
22q11.1–q11.21 (CES, *CECR2*)	0	3	2	2	·	·	4	VUS	P
3q29 (*DLG1*)	3	1	2	2	·	·	4	P	VUS
22q11.23 distal type III (F–G, *SMARCB1*)	3	1	·	·	·	4	4	—	VUS
15q13.1–q13.2 (BP3–BP4, *APBA2*)	1	0	1	1	·	1	3	VUS	VUS
22q11.21–q11.22 distal type I (D–E)	3	1	1	1	·	·	2	P	VUS
16p11.2 distal (BP2–BP3, *SH2B1*)	3	1	1	1	·	·	2	P	VUS
2q13 distal (*BCL2L11*)	2	1	2	·	·	·	2	VUS	—
22q11.21 central (B–D, *CRKL*)	2	1	2	·	·	·	2	VUS	—
7q11.23 distal (*HIP1*)	2	1	1	·	·	·	1	VUS	—
8p23.1 (*GATA4*)	3	2	·	1	·	·	1	—	VUS
10q11.22–q11.23 (LCR C–D)	1	1	·	1	·	·	1	—	VUS
15q13.3 (BP4–BP5, *CHRNA7*)	3	1	1	·	·	·	1	P	—
15q13.3–q14 (BP5–BP6, *GREM1*)	1	0	1	·	·	·	1	VUS	—
17q11.2 (Type 2, *NF1*)	3	2	1	·	·	·	1	P	—
17q21.31 (*KANSL1*)	3	1	1	·	·	·	1	P	—
15q24.1–q24.2 (LCR A–C)	3	1	·	·	·	1	1	—	P
TOTAL	​	​	64	29	6	11	110	​	​

HI, ClinGen haploinsufficiency score; TS, ClinGen triplosensitivity score (40, dosage sensitivity unlikely); Post, postnatal; Pre, prenatal; P, pathogenic; VUS, variant of uncertain significance; B, benign; —, direction not observed. The Deletion and Duplication columns show the ACMG/ClinGen classification for each dosage direction in that region. Counts are event-based (110 CNVs in 109 carriers). Region labels and coordinates follow Supplementary Table S1.

We categorized referral indications into six groups: neurodevelopmental/neurological, congenital/dysmorphic/sensory, growth/head size, syndromic and familial, reproductive/sexual development, and other systemic/miscellaneous. Each proband was assigned to a single primary indication, and these categories collectively represented the full postnatal denominator (Table 3). Neurodevelopmental referrals formed the largest group and accounted for 38/66 pathogenic recurrent-CNV carriers (57.58%), the largest contribution among indication groups. Pathogenic recurrent-CNV yield was similar across indication groups, ranging from 2.94% to 6.52%, with broadly overlapping confidence intervals that preclude ranking any single indication as higher-yield. Table 3 reports pathogenic recurrent-CNV carrier yield, whereas Figure 2 shows the event-based regional composition of all recurrent CNVs by referral indication. Yields also differed by test purpose, being much higher for targeted requests than in phenotype-driven testing (Table 4).

**TABLE 3:** Pathogenic recurrent-CNV frequency by referral-indication group in all postnatal probands.

Referral indication group	Probands	Pathogenic recurrent CNV	Yield (%)	95% CI
Neurodevelopmental and neurological disorders	952	38	3.99	2.92–5.43
Congenital, dysmorphic and sensory anomalies	374	11	2.94	1.65–5.19
Growth and head-size disorders	66	3	4.55	1.56–12.53
Syndromic and familial indications	159	9	5.66	3.01–10.41
Reproductive and sexual development disorders	64	2	3.13	0.86–10.70
Other systemic and miscellaneous indications	46	3	6.52	2.24–17.5
TOTAL	1661	66	3.97	3.14–5.02

**FIGURE 2:**
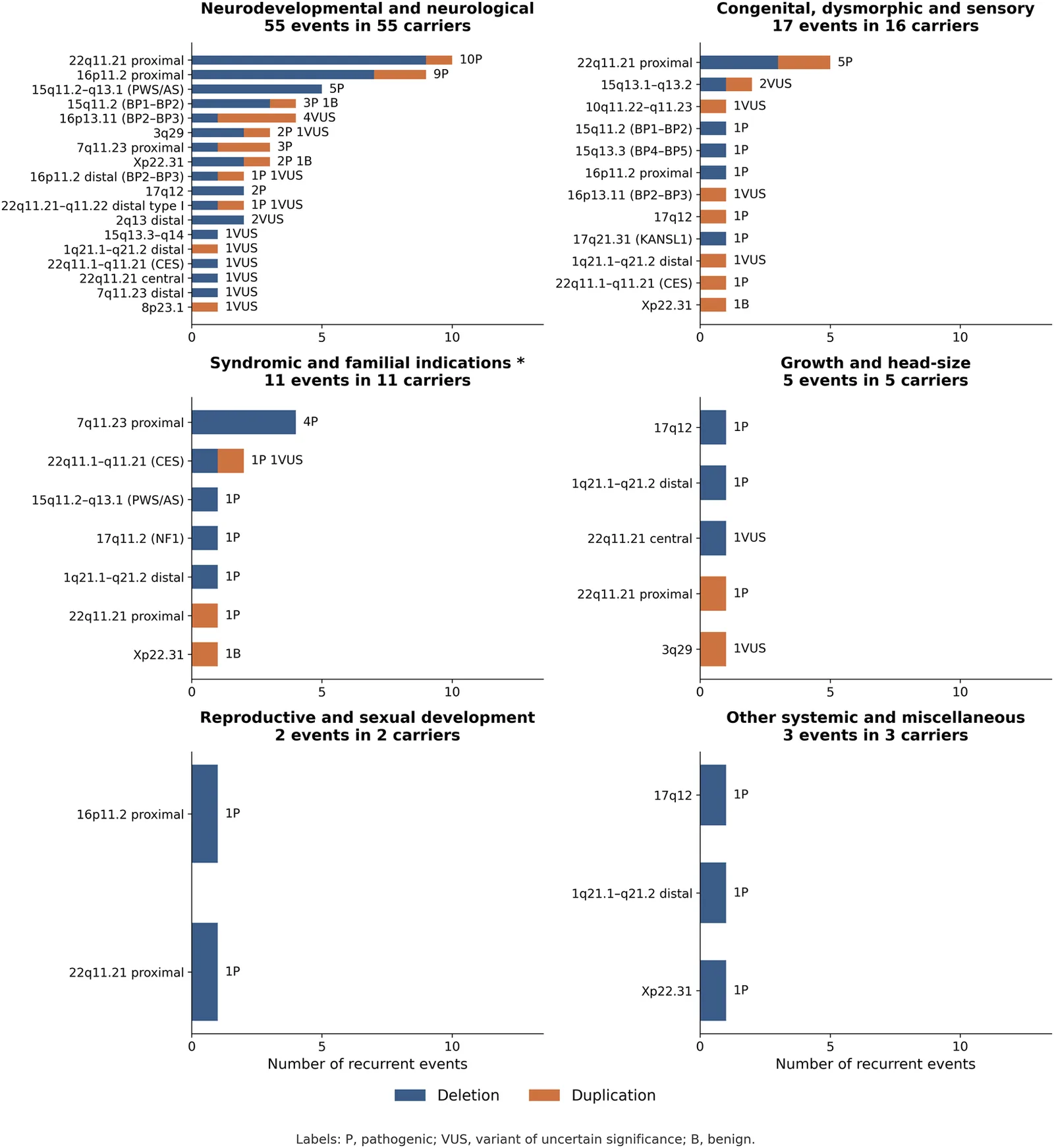
Recurrent CNV events by referral indication (postnatal). Bars are stacked by deletion (blue) and duplication (orange); labels give the ACMG breakdown (P, pathogenic; VUS, variant of uncertain significance; B, benign). 93 events in 92 carriers; one carrier harbored two independent recurrent CNVs. *This referral-indication group includes cases in which a specific syndrome or a familial variant prompted the request; its regional composition is therefore not an unbiased diagnostic sample.

**TABLE 4:** Pathogenic recurrent-CNV frequency by the purpose for which the array was requested (postnatal probands).

Test purpose	Requests	Pathogenic recurrent CNV	Yield (%)	95% CI
Diagnostic (phenotype-driven, hypothesis-free)	1,589	58	3.65	2.83–4.69
Targeted (specific recurrent CNV syndrome suspected)	19	6	31.58	15.36–53.99
Confirmatory (CNV already identified)	13	2	15.38	4.33–42.24
Characterizing (known karyotype abnormality)	40	0	0.0	0.0–8.76
TOTAL	1661	66	3.97	3.14–5.02

## 4 Discussion

Anchoring recurrent-CNV calls to ClinGen-curated regions and preserving dosage direction allowed us to quantify canonical recurrent CNVs as a distinct and substantial component of constitutional CMA reporting. Applying this framework, we found that recurrent CNVs accounted for 66/278 (23.74%) of postnatal and 7/28 (25.00%) of prenatal non-aneuploid pathogenic/likely pathogenic copy-number findings when only pathogenic recurrent CNVs were counted, indicating that aggregate reporting alone may make the contribution of canonical recurrent loci less visible. Notably, when each recurrent CNV was classified individually under the 2019 ACMG/ClinGen standards, pathogenic/likely pathogenic CNVs were found in only 66/92 postnatal (71.74%) and 7/17 prenatal recurrent-CNV carriers (41.18%); the remaining findings were of uncertain significance or benign. This directly quantifies the central observation that a recurrent-region label alone does not equate to pathogenicity.

The postnatal recurrent-CNV rate of 92/1,661 (5.54%) exceeded the 0.48% live-birth estimate from the MoBa cohort, as expected, given that this study focused on a tertiary diagnostic group enriched for neurodevelopmental, congenital, dysmorphic, growth-related, and syndromic/familial referral indications (Smajlagić et al., 2021; Perovic et al., 2022). Population prevalence and diagnostic yield therefore capture different aspects of the recurrent-CNV burden, and even well-characterized disorders such as Williams–Beuren and 22q11.2 deletion syndromes may be underrepresented in population cohorts owing to variable expressivity, incomplete penetrance, ascertainment bias, and other methodological differences (Smajlagić et al., 2021; Goh et al., 2025). In our postnatal cohort, pathogenic recurrent CNVs represented 23.74% of all pathogenic findings. Postnatal series applying the same ACMG framework but a broader recurrent definition, one that also includes terminal and single-gene microdeletion/duplication syndromes, have reported higher proportions: 21/35 (60.0%) in Miclea et al., 76/127 (59.8%) in Hu et al., and a similar predominance of recurrent over non-recurrent pathogenic CNVs in Wu et al. (Hu et al., 2019; Miclea et al., 2022; Wu et al., 2023). At the cohort level, our pathogenic recurrent-CNV yield of 66/1,661 (3.97%) was below the 6.6% and 6.1% reported in the neurodevelopmental cohorts of Wayhelová et al. and Cheng et al., respectively (Cheng et al., 2019; Wayhelova et al., 2019). Within our neurodevelopmental/neurological referral group, the yield was 38/952 (3.99%), close to the overall postnatal estimate and still below both reported rates. Differences in the definition of “recurrent” may contribute to this discrepancy. For example, Wayhelová et al. included 1q43–q44 and 22q13.3 deletion syndromes in their recurrent category, whereas our analysis was restricted to canonical NAHR-mediated intervals curated by ClinGen as recurrent. Notably, despite these quantitative differences, the predominance of deletions over duplications (approximately two-thirds) was consistent across all series, supporting a shared mechanistic basis for recurrent structural variation. When variants of uncertain significance were excluded, the overall non-aneuploid pathogenic or likely pathogenic yield of 16.74% fell within the expected 15%–20% range for referred postnatal CMA cohorts (Miller et al., 2010).

In our prenatal cohort, ClinGen-defined recurrent CNVs were detected in 17/401 samples (4.24%), with pathogenic recurrent CNVs detected in 7/401 (1.75%). Han et al., who defined recurrence by complete or partial overlap with DECIPHER-listed microdeletion/microduplication syndromes but classified variants under the same ACMG/ClinGen framework, reported an almost identical pathogenic recurrent-CNV rate of 134/7,645 (1.75%) and a total recurrent-CNV rate of 2.1% (Han et al., 2025). Kang et al. reported pathogenic recurrent-CNV frequencies of 83/7,078 (1.17%) overall and 40/1,781 (2.25%) in their fetal ultrasound-anomaly group (Kang et al., 2024). Although both studies screened overlapping sets of recurrent loci, their CNV inclusion criteria differed. Kang et al. included CNVs that encompassed or partially overlapped a recurrent region, provided that the region’s corresponding ClinGen HI or TS score was 1, 2, or 3. In our ultrasound-anomaly subgroup, the pathogenic recurrent-CNV yield was 6/348 (1.72%; 95% CI 0.79–3.71). These estimates provide descriptive context rather than evidence of equivalent diagnostic yield, given differences in CNV inclusion and classification, referral grouping, and cohort size. In the comparison with Han et al., dosage distribution also differed: their prenatal recurrent CNVs were deletion-predominant (66%), whereas duplications accounted for 11/17 recurrent CNVs (64.71%) in our cohort, several of which had low or uncertain penetrance and were classified as VUS rather than pathogenic. In this series, the pathogenic fraction was lower among prenatal duplications than deletions, consistent with the observation that a recurrent-region label does not by itself establish diagnostic certainty; the small number of prenatal carriers limits generalization of this distribution.

Test-purpose stratification is crucial because targeted, confirmatory, and phenotype-driven referrals have different clinical implications. For pathogenic recurrent CNVs, targeted requests had the highest observed per-test yield, 6/19 (31.58%), but contributed 6/66 carriers (9.09%). Phenotype-driven diagnostic testing had a lower yield, 58/1,589 (3.65%), but accounted for 58/66 carriers (87.88%), reflecting the much larger number of referrals in this group (Table 4). Additionally, since a “targeted” request may focus on recurrent CNV syndromes in one lab and on single-gene or other syndromes in another, yield comparisons across labs are not valid unless they specify request types.

Regional composition analysis adds another layer to the indication-based yield results. In neurodevelopmental cases, 22q11.21 proximal and 16p11.2 proximal stood out, while recurrent CNVs in the syndromic and familial referral-indication group most frequently involved 7q11.23 proximal (Williams–Beuren) and 22q11.1–q11.21 (CES) (Figure 2). Considering all recurrent-CNV carriers regardless of classification, 84/92 (91.30%) were identified through phenotype-driven diagnostic testing (without prior suspicion of a specific recurrent CNV syndrome), 6/92 (6.52%) through targeted requests and 2/92 (2.17%) through confirmatory testing. Of these 84 diagnostic carriers, 58 harbored pathogenic/likely pathogenic recurrent CNVs (Table 4); the remainder carried only VUS or benign recurrent CNVs. Genome-wide testing can identify CNVs associated with nonspecific phenotypes or variable expressivity, including recurrent CNVs with incomplete penetrance (Goh et al., 2025; 2026). Loci in the congenital and dysmorphic group are more dispersed, but small subgroup sizes require caution when interpreting these results.

The regional spectrum displayed clinical heterogeneity. One category comprised deletion-prone genomic disorders with well-described clinical phenotypes, such as 22q11.21 proximal, 7q11.23 proximal, 15q11.2–q13.1, 17q12 and 17q21.31 rearrangements (Gillentine et al., 2018). In our cohort, however, no syndrome-specific suspicion was recorded at referral for any of the 13 postnatal carriers of the proximal 22q11.21 deletion (Supplementary Table S2). A second category comprised reciprocal susceptibility loci such as 16p11.2 proximal, 1q21.1–q21.2 distal, 3q29, 16p13.11 (BP2–BP3) and 15q13.3 (BP4–BP5), where deletions and duplications are both possible but vary in penetrance, expressivity, and confidence in interpretation (Deshpande and Weiss, 2018). The third category consisted of low-penetrance or direction-sensitive duplications such as Xp22.31, 22q11.23 distal type III, and the benign 15q11.2 BP1–BP2 duplication (Goh et al., 2026). Only one of the 35 postnatal transmitting parents had a documented clinical diagnosis: the mother who transmitted a 3q29 deletion, who has attention-deficit/hyperactivity disorder, a recognized component of that phenotype (Sanchez Russo et al., 2021). Parental clinical information was obtained from referral records; parents were not clinically re-evaluated for this study. Reduced penetrance and variable expressivity complicate counseling on inherited findings (Rosenfeld et al., 2013; Maya et al., 2018). These results support reporting recurrent CNVs based on both locus and dosage direction, rather than solely on region name.

Pooled penetrance estimates are derived from case–control and population studies with their own ascertainment criteria and phenotype definitions; we therefore used them only as literature-based context for regional interpretation, not as estimates applicable to the present diagnostic cohort. This direction-specific interpretation is informed by ClinGen dosage sensitivity scores and by Goh et al. (2025), who reported higher penetrance for deletions than for reciprocal duplications at several loci represented here, including 22q11.2, 17q12 and distal 1q21.1. ClinGen likewise provides stronger haploinsufficiency than triplosensitivity evidence at some loci; triplosensitivity is considered unlikely for the Xp22.31 and 15q11.2 BP1–BP2 regions, and the corresponding duplications were classified as benign in this cohort. Published penetrance estimates also depend on how the phenotype is defined and how penetrance is calculated. For example, Goh et al. (2025) reported a pooled penetrance estimate of 51% for *BCR*/*MAPK1*-containing distal 22q11.2 duplications, whereas their subsequent analysis, using a revised definition and formula focused on intellectual disability, yielded an estimate of 24% (Goh et al., 2026). More broadly, the later study reported lower estimates for many low-to moderate-penetrance CNVs and discussed proposals against prenatal reporting of distal 16p11.2 duplications. This evolving evidence has broader implications for recurrent-CNV interpretation. Selected findings in our cohort illustrate distinct issues: uncertain phenotypic prediction for inherited prenatal 22q11.23 distal type III (F–G) duplications, and debated prenatal reportability of distal 16p11.2 (BP2–BP3) duplications, represented here by a single VUS. These examples illustrate why clinical interpretation depends on evidence specific to the genomic interval and phenotype, rather than on the recurrent-region label alone. These literature-based estimates were not used to assign pathogenicity; ACMG/ClinGen scoring remained the basis for classification throughout (Riggs et al., 2020). Clinical implications can differ even when both dosage states are classified as pathogenic: proximal 22q11.2 deletions cause DiGeorge/velocardiofacial syndrome, whereas reciprocal duplications show variable expression and are often inherited (Xue et al., 2021). For patient-specific clinical interpretation, region, dosage direction, inheritance, and phenotype concordance should be considered alongside the assigned classification, particularly for inherited duplications and reduced-penetrance loci in the prenatal setting.

Within the prenatal cohort, 5/6 *de novo* findings (83.33%) and 2/11 inherited findings (18.18%) were pathogenic, while 9/11 inherited findings (81.82%) were of uncertain significance or benign. These distributions are descriptive and based on small numbers. At the individual-case level, CNV–phenotype concordance varied. Discordant examples included a 15q11.2 BP1–BP2 deletion in a fetus with omphalocele (F005), a proximal 16p11.2 deletion in a fetus with myelomeningocele and Chiari II malformation (F008), a phenotype outside the craniocervical and posterior fossa spectrum described at this locus (Owen et al., 2018), and a benign Xp22.31 duplication in a fetus with shortened long bones (F017). Concordant examples included the 22q11.2 deletion with conotruncal and nuchal findings (F010), the distal 1q21.1 deletion with left-sided cardiac hypoplasia (F004), the 17q12/*HNF1B* deletion with the renal phenotype (F009) (Verscaj et al., 2024), and the Xp22.31/*STS* deletion detected through low maternal estriol (F015) (Han et al., 2026). A pathogenic classification does not, by itself, establish that a CNV explains the fetal ultrasound findings; the inherited 22q11.23 distal type III duplications (F011–F014) illustrate the converse, where recurrence risk is clear but outcome is not (Mary et al., 2022). Prenatal clinical interpretation should therefore consider the assigned classification alongside fetal imaging, inheritance, dosage direction, and region-specific penetrance.

A key practical takeaway from this study is that reporting of recurrent CNVs can be standardized beyond local naming conventions. By anchoring each call to a ClinGen-curated region, maintaining dosage direction, and recording inheritance, retrospective lab results can be harmonized into a more consistent cytogenomic dataset. This is especially important for adjacent or partially overlapping recurrent intervals, such as 16p11.2 proximal (BP4–BP5; 12 carriers, all pathogenic) versus 16p11.2 distal (BP2–BP3; 2 carriers, including a VUS duplication), and the 22q11.21 proximal (DiGeorge) region versus the 22q11.21 (CES), 22q11.21 central and 22q11.23 distal regions, where imprecise naming can obscure clinically relevant differences. Implementing a structured reporting approach that includes the reference recurrent region, dosage direction, the assigned ACMG/ClinGen classification, ClinGen haploinsufficiency/triplosensitivity evidence, inheritance, and phenotype concordance is particularly important for prenatal findings and low-penetrance or inherited events.

### 4.1 Limitations

This study has several limitations. Its retrospective, single-center design reflects local referral patterns, testing resources, and clinical triage practices. The observed recurrent-CNV frequencies are specific to this clinical CMA referral cohort and cannot be used to estimate population prevalence. The postnatal and prenatal cohorts differed in size, study period, and ascertainment, so they are described in parallel rather than treated as equivalent diagnostic populations; observed differences, including dosage-related patterns, should be interpreted as descriptive and hypothesis-generating.

We used published penetrance estimates only for interpretive context, because we could not estimate penetrance from this cohort, which lacks a control population. Subgroup analyses were constrained by small denominators, particularly for targeted, confirmatory, and smaller referral categories, resulting in wide confidence intervals, so yields by indication should not be read as phenotype-specific estimates. Referral groups were based on the primary reason for testing rather than systematic organ-specific phenotyping, limiting phenotype-specific interpretation.

The restriction to ClinGen-curated recurrent regions enabled a standardized audit but excluded non-recurrent pathogenic CNVs, so the full diagnostic CMA spectrum is not described. ClinGen dosage curations may also evolve over time, and the analysis reflects the 28 April 2026 release. The study spans two array platforms and a long diagnostic period; although canonical recurrent regions are less likely to be affected by platform differences, changes in resolution, reporting thresholds, and referral practice may have influenced the broader pathogenic/likely pathogenic CNV spectrum. Systematic clinical follow-up, pregnancy outcomes, and postnatal outcomes were unavailable for prenatal cases. We derived parental phenotype from referral records rather than systematic clinical assessment, and affected transmitting parents may therefore be underestimated in the postnatal cohort. Finally, patients investigated exclusively by FISH or MLPA were outside the scope of this CMA-based study. Such testing pathways may therefore reduce the representation of clinically recognizable recurrent syndromes in a CMA-based series.

## Data Availability

The original contributions presented in the study are included in the article/Supplementary Material, further inquiries can be directed to the corresponding author.

## Supplementary material

SUPPLEMENTARY TABLE S1 The 48 ClinGen-curated recurrent regions used in this study, with ISCA identifiers, GRCh38 coordinates, haploinsufficiency/triplosensitivity scores, region labels and pooled penetrance estimates.

SUPPLEMENTARY TABLE S2 CNV-level and case-level data for all recurrent CNV carriers (110 events in 109 carriers), including ACMG/ClinGen dosage scores, evidence codes and final classification.
